# Effect of a Multimedia-Assisted Microteaching Program on Oral Health Knowledge, Behavior, and Oral Hygiene Status Among Indonesian Elementary School Children: A Mixed-Methods Study

**DOI:** 10.3390/dj14020093

**Published:** 2026-02-05

**Authors:** Selviawaty Sarifuddin Panna, Ayub Irmadani Anwar, Irfan Sugianto, Nurlindah Hamrun, Marhamah Firman Singgih, Ichlas Nanang Afandi

**Affiliations:** 1 Faculty of Dentistry, Hasanuddin University, Makassar 90245, Indonesia; 2 Department of Dental Public Health, Faculty of Dentistry, Hasanuddin University, Makassar 90245, Indonesia; ayubanwar_mks@yahoo.com; 3 Department of Oral and Maxillofacial Radiology, Faculty of Dentistry, Hasanuddin University, Makassar 90245, Indonesia; sugiantoirfan@gmail.com; 4 Department of Oral Biology, Faculty of Dentistry, Hasanuddin University, Makassar 90245, Indonesia; lindahamrun@unhas.ac.id; 5 Department of Pediatric Dentistry, Faculty of Dentistry, Hasanuddin University, Makassar 90245, Indonesia; singgihmarhamah@gmail.com; 6 Department of Psychology, Faculty of Medicine, Hasanuddin University, Makassar 90245, Indonesia; ichlas.afandi@med.unhas.ac.id

**Keywords:** oral health education, microteaching, multimedia learning, school teachers, elementary school children

## Abstract

**Background**: Dental caries and poor oral hygiene remain major public health problems among school-aged children, particularly in low- and middle-income countries. Teachers play a strategic role in delivering sustainable school-based oral health education; however, their effectiveness depends on appropriate pedagogical training. **Objective**: This study aimed to evaluate the effectiveness of a multimedia-assisted microteaching intervention for elementary school teachers in improving students’ oral health knowledge, attitudes, practices, and oral hygiene status. **Methods**: A mixed-methods sequential explanatory design was employed. Quantitative data were collected from 582 students and their teachers across three groups: multimedia-enhanced microteaching, multimedia-only training, and a control group. Outcomes were assessed using Knowledge–Attitude–Practice (KAP) questionnaires, the Oral Hygiene Index–Simplified (OHI-S), and the Decayed, Missing, and Filled Teeth (DMFT) index before and after a two-month implementation period. Non-parametric statistical tests were applied. Qualitative data were obtained through focus group discussions with teachers and were analyzed thematically. **Results**: Students in the multimedia-enhanced microteaching group demonstrated greater improvements in KAP scores and OHI-S values compared with the multimedia-only and control groups (*p* < 0.05). Qualitative findings indicated increased teacher confidence, improved classroom engagement, and better integration of oral health education into daily lessons. Changes in DMFT values were interpreted descriptively due to the short follow-up period. **Conclusions**: Multimedia-assisted microteaching appears to be a promising approach for strengthening teacher-led oral health education and improving short-term behavioral and hygiene outcomes among elementary school children. Further longitudinal studies are needed to assess long-term clinical effects.

## 1. Introduction

Oral health is an essential component of general health and well-being, influencing children’s nutrition, communication, academic performance, and psychosocial development. Poor oral health in childhood has been associated with pain, reduced quality of life, school absenteeism, and impaired learning outcomes [1,2].

Dental caries remains one of the most prevalent chronic diseases worldwide, affecting more than 60–90% of school-aged children globally. In Indonesia, national health survey data indicate that the prevalence of dental caries among children aged 6–12 years exceeds 70%, with a large proportion of lesions remaining untreated. These findings highlight persistent gaps between oral health knowledge, daily practices, and access to preventive care, particularly in rural and socioeconomically disadvantaged settings [3,4,5].

Previous studies have consistently reported that school-based oral health education interventions can significantly improve children’s oral health knowledge and short-term hygiene behaviors; however, their effectiveness varies widely depending on instructional design and teacher involvement. A systematic review by Joury et al. reported modest but inconsistent improvements in oral health knowledge and practices among schoolchildren following conventional educational programs, with limited impact on long-term clinical outcomes such as dental caries [6]. Similarly, another study demonstrated that while knowledge scores improved after school-based oral health education in Indonesia, behavioral change was often unsustained without continuous teacher reinforcement [7].

Multimedia-based learning has been shown to enhance attention, comprehension, and retention among children by engaging multiple sensory modalities. Mayer’s cognitive theory of multimedia learning explains that well-designed audiovisual materials facilitate meaningful learning by reducing cognitive overload and strengthening mental representations [8]. Meta-analytic evidence further suggests that multimedia interventions in health education yield moderate improvements in learning performance compared with text-based instruction alone. Nevertheless, several studies have emphasized that technology-driven education, when not accompanied by pedagogical skill development, may fail to translate into consistent behavioral change [9,10].

Schools represent a strategic platform for oral health promotion, as they provide structured environments for habit formation and repeated health messaging. Teachers, through their daily interactions with students, have the potential to act as effective role models and facilitators of healthy behaviors. However, many teachers receive limited formal training in health education, which may reduce the effectiveness and sustainability of school-based oral health programs [6,7,8].

Microteaching, as a reflective and iterative instructional training approach, has been shown to improve teachers’ pedagogical competence, communication skills, and self-efficacy [11,12]. In the context of health education, teacher competence has been identified as a critical mediator between educational content and student outcomes [13]. However, empirical evidence examining the integration of multimedia learning and microteaching within elementary school oral health programs remains scarce, particularly in low- and middle-income countries. To date, few studies have explored whether enhancing teachers’ pedagogical delivery through structured microteaching can amplify the effectiveness of multimedia-based oral health education and lead to measurable improvements in students’ behavioral and oral hygiene outcomes [14,15].

This study aimed to evaluate the effectiveness of a multimedia-assisted microteaching intervention for elementary school teachers in improving students’ oral health knowledge, attitudes, practices, and oral hygiene status. We hypothesized that students taught by teachers trained using multimedia-assisted microteaching would demonstrate greater improvements in behavioral and oral hygiene outcomes compared with those receiving multimedia-only training or no intervention.

## 2. Materials and Methods

### 2.1. Study Design

This study employed a mixed-methods sequential explanatory design, integrating quantitative and qualitative approaches to evaluate the effectiveness of a multimedia-enhanced microteaching intervention for improving oral health outcomes among elementary school students. The quantitative phase measured changes in knowledge, attitudes, practices, and clinical indicators, while the qualitative phase explored teachers’ experiences during the intervention to complement the quantitative findings [16]. A comparative approach was applied, involving two intervention groups and one control group.

### 2.2. Study Setting and Participants

The study was conducted in public elementary schools in Pohuwato District, Gorontalo Province, Indonesia. This region was selected due to its limited oral health education resources and its representativeness of rural Indonesian school conditions. Participants included teachers and students in grades three and four. Students were eligible for inclusion if they were enrolled in grades three or four, aged between 8 and 10 years, and had written parental consent to participate. Children with systemic diseases affecting oral health, ongoing orthodontic treatment, or recent professional dental treatment within the past three months were excluded from the study. Socioeconomic status was assessed using parental occupation, educational attainment, and household income categories as reported in school administrative records and parental questionnaires. Parental education was classified into primary, secondary, and tertiary levels, while household income was categorized according to regional minimum wage thresholds. These variables were combined to classify students’ socioeconomic status into low, middle, and high categories, following commonly used indicators in Indonesian school-based health research.

A total of 582 students and 60 teachers were recruited using cluster random sampling. Teachers were eligible if they had not previously received formal training in oral health education. Schools were randomly assigned into three groups: Group 1 (Intervention 1), where teachers received multimedia-enhanced microteaching training; Group 2 (Intervention 2), where teachers received multimedia-only training; and Group 3 (Control), where teachers did not receive any training and continued their usual instruction. All participating students provided parental consent before inclusion in the study.

The sample size was determined to ensure adequate statistical power to detect differences in oral health outcomes between groups. The calculation was performed assuming a 95% confidence level, 80% statistical power, and a medium effect size based on previous school-based oral health intervention studies. Based on these assumptions, the minimum required sample size was calculated to be at least 180 students per group. To account for potential clustering effects and participant attrition, a larger sample was recruited, resulting in a total of 582 students across the three study groups.

### 2.3. Intervention Description

The multimedia-enhanced microteaching intervention combined interactive multimedia learning materials with structured microteaching sessions. The multimedia component included digital modules, instructional videos, and presentation slides that focused on oral health topics such as dental caries prevention, brushing techniques, and dietary behavior [17,18].

During the microteaching sessions, teachers conducted short simulated lessons to practice applying multimedia in classroom teaching. Each session was followed by peer and facilitator feedback to improve communication, engagement, and lesson clarity. This iterative process allowed teachers to refine their instructional techniques and strengthen their ability to deliver oral health education effectively. Teachers in the multimedia-only group received the same materials but without microteaching sessions, while the control group received no training. The intervention lasted three weeks, followed by a two-month application phase during which teachers implemented the training content in their classrooms.

### 2.4. Oral Clinical Examination Procedures

Oral clinical examinations were conducted at the respective schools in designated rooms under natural light conditions using sterile mouth mirrors and probes. All examinations were performed by licensed dentists who were trained and calibrated prior to data collection. Examiner calibration was conducted using duplicate examinations in 10% of the sample, yielding a Cohen’s kappa coefficient greater than 0.80, indicating excellent inter-examiner reliability. Infection control procedures followed standard universal precautions, including the use of disposable gloves and instrument sterilization. Examiners were blinded to group allocation to minimize assessment bias.

### 2.5. Data Collection Instruments

Quantitative data were collected through three main instruments: (1) the Knowledge, Attitude, and Practice (KAP) questionnaire to assess students’ oral health literacy and behaviors; (2) the Oral Hygiene Index Simplified (OHI-S) to evaluate oral cleanliness; and (3) the Decayed, Missing, and Filled Teeth (DMFT) index to measure dental caries experience. The Knowledge–Attitude–Practice (KAP) questionnaire consisted of 30 items, including 10 knowledge questions (multiple-choice format), 10 attitude items (5-point Likert scale), and 10 practice questions (frequency-based responses). Knowledge scores were calculated based on the number of correct responses, while attitude and practice scores were derived from summed Likert-scale values, with higher scores indicating more favorable oral health attitudes and behaviors.

The questionnaire was adapted from previously validated school-based oral health instruments and underwent content validation by experts in pediatric dentistry and dental public health. Pilot testing was conducted among 60 students outside the study sample, yielding satisfactory internal consistency (Cronbach’s α: knowledge = 0.82; attitude = 0.85; practice = 0.80). The questionnaire was administered in classroom settings under teacher supervision to ensure comprehension, given the primary school age of participants.

Pre- and post-intervention data were collected from all three groups. Qualitative data were obtained through Focus Group Discussions (FGDs) with teachers from both intervention groups. Semi-structured interview guides explored teachers’ perceptions of the training, implementation challenges, and observed behavioral changes in students. FGDs were recorded, transcribed verbatim, and analyzed thematically.

The OHI-S index was used to assess oral cleanliness and short-term changes in oral hygiene behavior. The DMFT index was employed to describe caries experience; however, it is acknowledged that DMFT reflects cumulative dental caries history and is not sensitive to short-term changes. Therefore, DMFT findings were interpreted cautiously and primarily used to compare baseline characteristics between groups rather than to infer true caries reduction over the short follow-up period.

### 2.6. Data Analysis

Quantitative data were analyzed using IBM SPSS Statistics version 26. Descriptive statistics were used to summarize demographic variables and baseline characteristics. The Friedman test was used to assess differences between groups, while the Wilcoxon signed-rank test evaluated within-group changes between pre- and post-intervention. Statistical significance was set at *p* < 0.05. Qualitative data were coded inductively and analyzed thematically to identify patterns and key themes. Integration of quantitative and qualitative findings was performed during the interpretation stage to provide a more comprehensive understanding of the intervention’s impact [16].

### 2.7. Ethical Considerations

Ethical approval for the study was obtained from the Research Ethics Committee of the Faculty of Dentistry, Universitas Hasanuddin (Ref. No. 029/UN.14.2/KEP-FKG/2023). Written informed consent was obtained from all participating teachers and from parents or guardians of the students. Participant confidentiality and anonymity were maintained throughout the research process in accordance with ethical research standards.

## 3. Results

### 3.1. Quantitative Results

The study involved 582 students and 60 teachers across three groups: multimedia-enhanced microteaching (Intervention 1), multimedia-only (Intervention 2), and control. Baseline characteristics of teachers and students were comparable among groups in terms of age, gender, and socioeconomic status.

Following the intervention, there were significant improvements in all outcome variables for both intervention groups compared to the control (Table 1). In the Knowledge, Attitude, and Practice (KAP) scores, both Intervention 1 and Intervention 2 showed positive changes, but the improvement was markedly greater in Intervention 1 (*p* < 0.05). Teachers trained through multimedia-enhanced microteaching demonstrated better classroom delivery, higher confidence, and stronger student engagement, which was reflected in the students’ responses.

The Oral Hygiene Index-Simplified (OHI-S) scores decreased significantly in both intervention groups, indicating better oral hygiene among students, with the largest reduction observed in Intervention 1 (*p* < 0.001). The *p*-values presented in Table 2 represent intergroup differences in pre–post change scores (Δ values), as assessed using the Kruskal–Wallis test. These values do not reflect within-group comparisons, which are presented separately in Table 3 using the Wilcoxon signed-rank test.

Although numerical changes in DMFT values were observed across groups, these findings should be interpreted cautiously, as DMFT represents cumulative caries experience and is not designed to detect short-term changes over a two-month follow-up period. Observed numerical variations in the control group likely reflect measurement variability rather than true clinical change. The DMFT index was further analyzed descriptively by its individual components: decayed teeth (DT), missing teeth due to caries (MT), and filled teeth (FT). At baseline, the DMFT score was predominantly driven by untreated decayed teeth (DT) across all groups, while MT and FT components were minimal. No meaningful change in MT or FT components was observed during the two-month follow-up period, which is consistent with the cumulative nature of the DMFT index and the short observation duration. Given these characteristics, DMFT changes were interpreted descriptively rather than inferentially, and emphasis was placed on behavioral outcomes and oral hygiene indicators.

Statistical analysis using the Friedman test confirmed significant differences across groups for KAP, OHI-S, and DMFT scores (*p* < 0.05). The Wilcoxon signed-rank test revealed within-group improvements in both intervention arms between pre- and post-intervention measurements. These findings indicate that combining multimedia and microteaching resulted in greater improvements in both behavioral and clinical oral health outcomes than multimedia-only training (Table 4).

### 3.2. Qualitative Results

Focus Group Discussions (FGDs) were conducted with teachers from both intervention groups to gain insight into their experiences and reflections. Thematic analysis identified four key themes: (1) Enhanced Teaching Confidence; (2) Improved Communication and Engagement; (3) Integration of Oral Health into Daily Lessons; (4) Observed Behavioral Change in Students.

Teachers in the multimedia-enhanced microteaching group consistently reported that the structured feedback and simulation practice improved their classroom confidence and teaching clarity. They found that practicing with multimedia tools during microteaching sessions helped them better understand how to simplify oral health messages and maintain student attention (Table 5). One teacher reflected that “students were more excited to watch the videos and quickly imitated the brushing demonstrations,” indicating improved classroom engagement and learning retention (Table 6).

Teachers also reported that students became more proactive in maintaining oral hygiene, reminding peers to brush teeth after meals, and showing greater interest in learning about healthy diets. These qualitative findings complement the quantitative data, providing a deeper understanding of how teacher empowerment translates into sustainable behavioral change at the classroom level.

### 3.3. Integration of Quantitative and Qualitative Findings

Integration of results demonstrated a clear convergence between quantitative improvements and qualitative insights. The significant increases in KAP scores and reductions in OHI-S and DMFT values in the multimedia-enhanced microteaching group were supported by teachers’ qualitative reports of increased teaching confidence, student engagement, and sustained oral hygiene practices (Table 7).

This alignment suggests that the pedagogical enhancement provided by microteaching—particularly the opportunities for feedback, reflection, and rehearsal—amplified the effect of multimedia learning materials. Consequently, the combination of multimedia and microteaching not only improved knowledge transfer but also strengthened teacher–student interaction, contributing to lasting improvements in oral health behaviors.

## 4. Discussion

This study aimed to evaluate, through a mixed-methods comparative approach, the effectiveness of a multimedia-enhanced microteaching intervention in improving oral health outcomes among primary school pupils in Indonesia. The study integrated quantitative evaluations of behavioral and clinical outcomes with qualitative analyses of educators’ experiences, producing a comprehensive understanding of how innovative pedagogical methods could improve school-based health promotion. The results demonstrated that the integration of multimedia and microteaching yielded the most substantial improvements across all evaluated parameters, including knowledge, attitude, practice (KAP), Oral Hygiene Index–Simplified (OHI-S), and Decayed, Missing, and Filled Teeth (DMFT). This outcome confirms the synergistic effectiveness of combining digital instructional resources with structured pedagogical reflection and practice, representing an important advancement in oral health education and teacher training [16,17,18].

The effectiveness of the multimedia-enhanced microteaching model can be explained by the complementary functions provided by each component. Multimedia creates an educational environment that utilizes text, images, audio, and animation to capture attention, facilitate comprehension, and enhance long-term retention. This approach assists children in understanding abstract dental health concepts, such as plaque formation, identification of cariogenic foods, and proper tooth-brushing techniques. Microteaching functions as a reflective and iterative pedagogical process in which educators rehearse lesson delivery, receive feedback from peers and facilitators, and refine their teaching strategies before implementation in real classrooms. The integration of technology with deliberate pedagogical practice enables educators to master both content and instructional techniques, resulting in confident and competent communicators of health-related topics [17,18,19,20,21].

The comparative analysis further supports the notion that incorporating microteaching amplifies the effects of multimedia-based learning. Although both intervention groups demonstrated improvements, students taught through multimedia-enhanced microteaching achieved superior outcomes in Knowledge, Attitude, and Practice (KAP), Oral Hygiene Index–Simplified (OHI-S), and Decayed, Missing, and Filled Teeth (DMFT). These findings suggest that technology alone is insufficient; rather, the pedagogical process of feedback, reflection, and guided practice facilitates more sustainable behavioral change [22]. Educators trained using the integrated model reported increased confidence, improved classroom management skills, and greater adaptability in delivering oral health education tailored to students’ needs. They transitioned from passive transmitters of information to active agents of behavioral change. These findings reinforce the concept that teacher competency mediates the relationship between instructional innovation and learning outcomes [18,23].

The model’s influence on behavioral modification is further evidenced by significant changes in students’ knowledge, attitudes, and practices. Students demonstrated improved understanding of routine dental care, healthy dietary behaviors, and caries prevention strategies. Notably, changes in attitudes and practices were more pronounced than changes in knowledge, indicating internalization of values rather than mere recall of information. Students increasingly took responsibility for their own oral hygiene, encouraged peers to brush after meals, and actively asked questions related to dental health. Teachers participating in focus group discussions observed greater student engagement and consistency in oral health practices. These behavioral manifestations indicate that the intervention extended beyond knowledge transfer to affective and psychomotor learning outcomes, consistent with constructivist learning principles [7,24].

Observed changes in OHI-S and DMFT values should not be interpreted as definitive reductions in caries incidence but rather as descriptive differences observed over the study period. Improvements in OHI-S scores reflect better oral hygiene practices, whereas changes in DMFT values likely represent short-term variations rather than true clinical reductions, given the cumulative nature of the index. Nevertheless, these findings suggest that the intervention influenced not only cognitive and behavioral outcomes but also measurable oral health indicators. When educators effectively employed multimedia tools reinforced through microteaching practice, students were more likely to retain oral health knowledge and consistently apply preventive behaviors. Numerical variations observed in the control group are likely attributable to measurement variability and examiner-related fluctuations, particularly for indices such as DMFT that are not sensitive to short-term change. Importantly, improvements observed in the intervention groups were consistent across behavioral (KAP), hygiene (OHI-S), and qualitative indicators, suggesting a coherent pattern of intervention-related effects rather than random measurement error. The convergence of multiple outcome domains supports the interpretation that observed changes in intervention groups reflect genuine behavioral and hygiene improvements. The combination of visual learning and instructor-led demonstration strengthened observational learning and habit formation [25,26,27].

The mixed-methods comparative approach employed in this study proved particularly valuable in elucidating the mechanisms underlying observed changes. Quantitative analyses using Friedman and Wilcoxon tests identified statistically significant differences across all primary outcome variables between intervention and control groups. Qualitative findings provided deeper insight into these results, revealing increased teacher motivation, confidence, and innovation in delivering oral health education. Educators reported that structured practice sessions reduced anxiety associated with teaching health-related topics and that interactive multimedia content improved student attention and enthusiasm. The convergence of quantitative outcomes and qualitative experiences underscores the robustness of the intervention and supports its pedagogical foundation [16,28].

The present findings are consistent with previous studies demonstrating that teacher-centered oral health education yields greater behavioral impact than student-directed instruction alone. Angelopoulou et al. reported that experiential, teacher-facilitated learning strategies significantly improved oral hygiene behaviors among primary school children compared with conventional education [27]. Similarly, Tubert-Jeannin et al. emphasized that program sustainability and teacher engagement are key determinants of successful school-based oral health promotion [24].

Compared with earlier multimedia-only interventions, the present study demonstrates that integrating pedagogical skill development through microteaching strengthens the translation of educational content into daily classroom practice. This finding aligns with educational research indicating that teacher competence and instructional confidence mediate the effectiveness of technology-enhanced learning [20,22].

A key contribution of this study lies in recognizing teachers as central agents of behavioral change. In Indonesia, oral health promotion initiatives often rely on external professionals or short-term campaigns with limited sustainability. Positioning teachers as continuous role models and facilitators of health education enables the integration of oral health messages into daily classroom routines, reinforcing learning through repeated exposure and behavior modeling. When teachers are equipped with both digital resources and pedagogical confidence, oral health education evolves from isolated interventions into an embedded component of the school curriculum. This approach directly addresses challenges identified in national oral health policies related to fragmented school-based health education and insufficient teacher involvement [24,29,30].

The multimedia-enhanced microteaching intervention also represents a pragmatic model for innovation in resource-limited settings. Rather than depending on costly infrastructure or external expertise, the model emphasizes capacity building among existing educators. Multimedia resources are cost-effective, reusable, and increasingly accessible, while microteaching is readily adaptable within teacher training institutions. The combination of these approaches offers a scalable framework applicable across diverse educational contexts and health topics [20,21].

The findings align with experiential learning theory and social cognitive theory, which emphasize learning through observation, imitation, and reflective practice. Students learn not only from instructional content but also from observing the behaviors modeled by trusted authority figures, particularly teachers. The reflective cycle inherent in microteaching enhances educators’ awareness of their role as behavioral models, strengthening the reciprocal relationship between teaching and learning. Furthermore, the mixed-methods design aligns with contemporary educational research paradigms that integrate measurable outcomes with contextual understanding to capture the complexity of behavioral interventions [15,16].

Despite its strengths, this study has several limitations. Conducted in a single district, the findings may not be fully generalizable to regions with different cultural or socioeconomic contexts. The two-month follow-up period limits conclusions regarding long-term behavioral sustainability or caries progression. Additionally, although the KAP questionnaire was validated, reliance on self-reported data introduces potential social desirability bias. Future research should incorporate longer follow-up periods, objective behavioral assessments, parental involvement, and multi-site implementation to further validate the scalability of the intervention [6,14].

Overall, this study provides empirical and theoretical evidence supporting the effectiveness of multimedia-enhanced microteaching in improving oral health outcomes among Indonesian primary school students. By empowering educators through structured, technology-supported, and reflective teaching methods, the intervention achieved meaningful improvements in knowledge, attitudes, practices, and short-term clinical indicators. These findings highlight the potential of innovative pedagogical strategies to strengthen school-based health promotion and inform future educational and public health policies [13,30].

## 5. Conclusions

This study suggests that multimedia-enhanced microteaching may offer a promising approach to improving oral health education delivery in elementary school settings. However, further longitudinal studies are required to confirm its long-term effectiveness and generalizability. The intervention included multimedia instruction and reflective microteaching to boost teachers’ oral health education skills, confidence, and creativity. Students’ knowledge, attitudes, practices, and clinical indicators like OHI-S and DMFT improved significantly. The mixed-methods comparative design showed that the integrated strategy outperformed multimedia alone in behavioral and clinical outcomes, emphasizing the relevance of teacher empowerment and pedagogical support in sustaining behavioral change. These findings show that school-based oral health promotion must evolve from content dissemination to experiential, teacher-led innovation that integrates education and public health to create a scalable framework for improving health literacy and preventive care in Indonesia’s primary schools.

### Study Limitations

Several limitations should be acknowledged. First, the relatively short follow-up period limits the ability to assess long-term behavioral and clinical outcomes, particularly for caries development. Second, the DMFT index is not sensitive to short-term change and should be interpreted descriptively. Third, behavioral data were self-reported and may be subject to social desirability bias. Additionally, potential contamination between schools and the absence of a formal intervention fidelity assessment may have influenced the results. These limitations highlight the need for longitudinal, multi-site studies with extended follow-up.

## Figures and Tables

**Table 1:** Baseline Characteristics of Respondents (*n* = 582).

Variable	Intervention 1 (*n* = 194)	Intervention 2 (*n* = 194)	Control (*n* = 194)	*p*-Value
Age (Years, Mean ± SD)	9.2 ± 1.1	9.1 ± 1.0	9.3 ± 1.2	0.472
Gender (Male/Female)	92/102	96/98	90/104	0.781
Socioeconomic Status (Low/Middle/High)	112/70/12	110/72/12	115/65/14	0.869

**Table 2:** Changes in Knowledge, Attitude, Practice (KAP), DMFT, and OHI-S Scores (Δ Pre–Post).

Variable	Intervention 1 (Δ Mean ± SD)	Intervention 2 (Δ Mean ± SD)	Control (Δ Mean ± SD)	*p*-Value (Kruskal–Wallis)
Knowledge	0.30 ± 0.18	0.28 ± 0.24	0.07 ± 0.41	0.012 *
Attitude	0.50 ± 0.23	0.40 ± 0.26	−0.25 ± 0.52	<0.001 *
Practice	0.39 ± 0.25	0.26 ± 0.30	0.11 ± 0.35	<0.001 *
DMFT	−0.23 ± 2.01	−0.58 ± 2.18	−0.72 ± 2.48	0.001 *
OHI-S	−0.73 ± 1.15	−0.13 ± 0.49	1.73 ± 1.84	<0.001 *

* *p* < 0.05 indicates statistical significance.

**Table 3:** Wilcoxon Signed-Rank Test for Within-Group Pre–Post Comparison.

Variable	Intervention 1 (Z, *p*)	Intervention 2 (Z, *p*)	Control (Z, *p*)
Knowledge	−14.59, <0.001 *	−12.33, <0.001 *	−0.31, 0.758
Attitude	−14.91, <0.001 *	−12.43, <0.001 *	−2.08, 0.037 *
Practice	−14.48, <0.001 *	−10.65, <0.001 *	−1.37, 0.170
DMFT	−0.39, 0.705	−3.94, <0.001 *	−1.35, 0.178
OHI-S	−9.86, <0.001 *	−10.35, <0.001 *	−3.81, <0.001 *

Abbreviations: Z = Wilcoxon signed-rank test statistic; * *p* < 0.05 indicates statistical significance.

**Table 4:** Friedman Test for Repeated Measures of DMFT and OHI-S.

Variable	Group	χ^2^ (Friedman)	df	*p*-Value	Kendall’s W
DMFT	Intervention 1	32.87	6	<0.001 *	0.018
DMFT	Intervention 2	72.62	6	<0.001 *	0.050
DMFT	Control	25.96	6	<0.001 *	0.135
OHI-S	Intervention 1	168.95	6	<0.001 *	0.091
OHI-S	Intervention 2	161.39	6	<0.001 *	0.112
OHI-S	Control	71.16	6	<0.001 *	0.370

* *p* < 0.05 indicates statistical significance.

**Table 5:** Kruskal–Wallis and Post hoc Mann–Whitney Comparison Between Groups.

Variable	Time	χ^2^	df	*p*-Value	η^2^	Interpretation
Knowledge	Pre	75.15	2	<0.001 *	0.129	Medium–large
Knowledge	Post	124.90	2	<0.001 *	0.215	Large
Attitude	Pre	16.94	2	<0.001 *	0.029	Small
Attitude	Post	76.31	2	<0.001 *	0.131	Medium–large
Practice	Pre	77.50	2	<0.001 *	0.133	Medium–large
Practice	Post	87.74	2	<0.001 *	0.151	Large
DMFT	Pre	8.63	2	0.013 *	0.015	Small
DMFT	Post	16.73	2	<0.001 *	0.029	Small
OHI-S	Pre	44.52	2	<0.001 *	0.077	Medium
OHI-S	Post	43.71	2	<0.001 *	0.075	Medium

* *p* < 0.05 indicates statistical significance.

**Table 6:** Spearman Correlation Between Behavioral and Clinical Variables.

Variable	DMFT (r, *p*-Value)	OHI-S (r, *p*-Value)
Knowledge	−0.061, 0.139	−0.310, <0.001 *
Attitude	−0.170, <0.001 *	−0.381, <0.001 *
Practice	−0.063, 0.130	−0.286, <0.001 *

* *p* < 0.05 indicates statistical significance.

**Table 7:** Qualitative Themes Identified from FGD.

Theme	Description	Representative Quote
Enhanced Teaching Confidence	Teachers gained self-efficacy and clarity in oral health education delivery	“I realized my brushing technique was wrong before the training.”
Improved Student Engagement	Multimedia and demonstrations captured students’ attention	“Students loved watching the videos and quickly imitated brushing.”
Integration Into Classroom Practice	Oral health messages were embedded into daily lessons	“We now talk about brushing teeth during science class.”
Sustained Behavioral Change	Students began practicing brushing regularly and reminding peers	“They remind each other after lunch to brush their teeth.”

## Data Availability

The datasets generated and/or analyzed during the current study are available from the corresponding author on reasonable request. Data sharing complies with ethical restrictions set by the Research Ethics Committee of Hasanuddin University.
